# Antimicrobial Susceptibility and Genetic Characterisation of *Burkholderia pseudomallei* Isolated from Malaysian Patients

**DOI:** 10.1155/2014/132971

**Published:** 2014-10-14

**Authors:** Yalda Khosravi, Kumutha Malar Vellasamy, Vanitha Mariappan, Shet-Lee Ng, Jamuna Vadivelu

**Affiliations:** Tropical Infectious Disease Research and Education Center (TIDREC), Department of Medical Microbiology, Faculty of Medicine, University of Malaya, 50603 Lembah Pantai, Kuala Lumpur, Malaysia

## Abstract

*Burkholderia pseudomallei*, the causative agent of melioidosis, is intrinsically resistant to many antibiotics. Ceftazidime (CAZ), the synthetic *β*-lactam, is normally used as the first-line antibiotic therapy for treatment of melioidosis. However, acquired CAZ resistance can develop *in vivo* during treatment with CAZ, leading to mortality if therapy is not switched to a different antibiotic(s) in a timely manner. In this study, susceptibilities of 81 *B. pseudomallei* isolates to nine different antimicrobial agents were determined using the disk diffusion method, broth microdilution test and Etest. Highest percentage of susceptibility was demonstrated to CAZ, amoxicillin/clavulanic acid, meropenem, imipenem, and trimethoprim/sulfamethoxazole. Although these drugs demonstrated the highest percentage of susceptibility in *B. pseudomallei*, the overall results underline the importance of the emergence of resistance in this organism. PCR results showed that, of the 81 *B. pseudomallei*, six multidrug resistant (MDR) isolates carried *bpeB*, *amrB*, and *BPSS1119* and *penA* genes. Genotyping of the isolates using random amplified polymorphic DNA analysis showed six different PCR fingerprinting patterns generated from the six MDR isolates clusters (A) and eight PCR fingerprinting patterns generated for the remaining 75 non-MDR isolates clusters (B).

## 1. Introduction

Melioidosis, an infectious disease of major public health importance, is a multifaceted disease which is difficult to treat and results in high morbidity and mortality. It is mostly endemic in Southeast Asia, Northern Australia, the Indian subcontinent, and Central and South America [1, 2]. The causative agent of this fatal disease,* Burkholderia pseudomallei*, is an environmental saprophyte that can be isolated from soil and water and is known as a potential biological warfare agent [2]. Infection of* B. pseudomallei* is acquired mainly through wound inoculation, inhalation, or ingestion [2, 3]. Melioidosis may arise many years after exposure, commonly in association with compromised immunity. Overall, the mortality rate of melioidosis is about 50% in Northeast Thailand (35% in children) [3] and 19% in Australia [4]. However, in Malaysia, it was associated with 65% mortality especially in the septicaemic form in the 1980s and reduced to 19–37% in the past 20 years [1, 5–7]. Recurrence of infection is the most important complication in survivors despite prolonged antimicrobial treatment and this has been reported in 10% of Thai patients who survived the primary infection episode [4].

*B. pseudomallei* is intrinsically resistant to many antibiotics, including penicillin, first- and second-generation cephalosporins, macrolides, rifamycins, colistin, and aminoglycosides, but is usually susceptible to amoxicillin/clavulanic acid (AMC), chloramphenicol (CL), doxycycline (DOXY), trimethoprim/sulfamethoxazole (TS), ureidopenicillins, ceftazidime (CAZ), and carbapenems [8]. CAZ, AMC, or the carbapenem antibiotics are used for the initial parenteral phase of the therapy, followed by a prolonged course of oral antimicrobial therapy with either TS with or without DOXY or AMC [9]. However, CAZ-resistant (CAZR) and/or AMC-resistant* B. pseudomallei* have emerged, ultimately leading to treatment failure [9]. The carbapenems have been reported to have good bactericidal activities against* B. pseudomallei* and have been used effectively to treat patients with septicaemic melioidosis [10, 11]. However, increased use of carbapenems may again give rise to resistance as observed with CAZ and AMC.

Owing to the difficulty in eradication of the organism following infection, prolonged antibiotic therapy is needed and a high rate of relapse was observed if the therapy is incomplete [12]. Furthermore, recurrence of infection is common despite adequate antimicrobial therapy [13]. It has been shown that* B. pseudomallei* isolated from relapse cases and persistent infections were resistant to antibiotics for treatment [3, 14].

Thus, the aim of this study was to investigate the antimicrobial susceptibility profile of* B. pseudomallei* against a panel of medically relevant antibiotics including CAZ, AMC, DOXY, and TS. Evaluation using other antimicrobials such as CL, meropenem (MERO), imipenem (IMP), tigecycline (TGC), and clarithromycin (CLA) were also included. The antimicrobial susceptibility was performed using two determination methods, that is, the agar disc diffusion and Etest. Additionally, polymerase chain reaction (PCR) technique utilising specific primers was also used for detection of antibiotics resistant genes as this proves to be reliable for detection of *β*-lactam, aminoglycosides, and CAZR genes in multidrug resistant (MDR) isolates. Further investigation using random amplification of polymorphic DNA (RAPD) analysis was performed to identify the genetic relationships among the isolates and the mode of dissemination of the antibiotic resistance genes.

## 2. Materials and Methods

### 2.1. Ethics Statement

Ethics approval is not required in this study. No consent was required since no human participant was involved. All 70 clinical* B. pseudomallei* isolates used in this study were obtained from old archival bacterial collection and all the isolates used were anonymised. Since our institute is a teaching hospital, bacterial isolates obtained as a part of a diagnostic screening are archived and such cases are exempted from obtaining ethical clearances. The study however has an Institutional Biosafety Committee approval.

#### 2.1.1. Bacterial Strains

A total of 81* B. pseudomallei* isolates (70 clinical, 10 animal, and 1 soil) and one* Burkholderia thailandensis* (ATCC 700388) isolates were investigated in this study. Of the 70 clinical* B. pseudomallei*, 60 isolates were obtained from archival collections of strains isolated at the Medical Microbiology Diagnostic Laboratory, University of Malaya Medical Centre (UMMC, Kuala Lumpur), and 10 from Hospital Tengku Ampuan Afzan (HTAA, Kuantan, Pahang). The ten animal isolates were kindly provided by S Nathan (Universiti Kebangsaan Malaysia), Malaysia, and the soil isolate was obtained from the Bacteriology Unit, Institute of Medical Research (IMR), Malaysia. These isolates were confirmed using Gram-stain, morphological appearance on Ashdown agar, molecular identification using an in-house PCR method [18] and standard biochemical characterisation using API 20NE assay (Bio-Merieux, France).* Escherichia coli* (ATCC 25922) was used as control while* B. pseudomallei* K96243 was used as reference strain. The isolates used in this study were obtained from different sources, that is, soil, animal and human (blood, pus, sputum, urine, spleen, and lungs).

### 2.2. Antibiotic Susceptibility Testing

#### 2.2.1. Disk Diffusion Test

*In vitro* antimicrobial susceptibility to nine antibiotics/antimicrobials, namely, CL, AMC, DOXY, MERO, IMP, CAZ, TGC, CLA, and TS, was determined using disc diffusion method according to the British Society of Antimicrobial Chemotherapy [19]. Nutrient agar plates were seeded with 100 *μ*L of 10^6^ colony forming unit per milliliter (CFU/mL) of the test isolates, which had been adjusted to 0.5 McFarland reading spectrophotometrically at 600 nm. The agar plates were then incubated at 37°C for 24 hrs.

#### 2.2.2. Minimum Inhibitory Concentrations (MICs)

Antimicrobial MICs were determined using the broth dilution method and Etest. The broth dilution method was performed using Luria-Bertani (LB) broth (Difco, Lennox) for 24 hrs at 37°C with 10^5^ CFU/mL of* B. pseudomallei* (previously washed using normal saline) as the inoculum. Interpretation of the broth dilution results was based on the NCCLS MIC breakpoints for non-*Enterobacteriaceae* and MIC for* B. pseudomallei* and* B. thailandensis* [19]. Inhibition of the bacterial growth was confirmed by spectrophotometric measurement at 600 nm and plating of the serial dilutions onto LB agar. Wells containing bacteria without antibiotics and fresh LB broth were included as positive and negative growth controls, respectively.* E. coli* ATCC 25922 was used as quality control organism in the antimicrobial MIC determinations. MIC determination using Etest strips (AB Biodisk, Sweden) was performed according to the manufacturer's instruction.

#### 2.2.3. Polymerase Chain Reaction (PCR)

The DNA of all the isolates tested was extracted using Qiagen Mini Amp Kit (Qiagen, USA) according to the manufacturer's instruction. Primers (PenA-F and PenA-R) were used to identify the presence of* penA* gene from class A *β*-lactamase which causes the CAZR (Table 1). Presence of the efflux pump, another main resistance mechanism, was also detected using the degenerate primers, MxBs, MxYs, and MxFs (Table 1). PCR amplification was performed in 25 *μ*L mixture containing 1X PCR buffer (MBI Fermentas, USA), 2 mM MgCl_2_, 0.2 mM dNTP (MBI Fermentas, USA), 2.5 U* Taq* DNA polymerase (MBI Fermentas, USA), and 2 *μ*L of DNA template. The PCR conditions used were as follows: initial denaturation at 95°C for 5 mins, 30 cycles of 95°C for 1 min, 55°C for 30 s and 72°C for 1 min, followed by a final extension for 10 mins at 72°C. The amplified PCR products were analysed using 1.5% agarose gel (Promega, USA) electrophoresis in 1X TBE buffer at 90 V for 1 hr and visualised using 3 *μ*L SYBR safe (Life Technology, USA) staining under UV illumination. The limit of dilution was determined by subjecting the DNA of the targeted organisms to PCR after 10-fold serial dilution to produce DNA concentration ranging from 10 mg/mL to 10 fg/mL [17].

#### 2.2.4. Genotypic Analysis Using Random Amplified Polymorphic DNA (RAPD)-PCR

The RAPD analysis was performed using primer 272 (Table 1) in order to determine the fingerprinting patterns of the* B. pseudomallei* isolates [20]. The RAPD-PCR mixture (25 *μ*L) consisted of 100 ng of genomic DNA, 0.5 *μ*M primer, 1.25 U of Taq polymerase (MBI Fermentas), 0.2 mM of dNTP (Fermentas, USA), 1X Taq buffer with KCl (pH8), and MgCl_2_ (2 mM). The cycling condition employed includes (i) 4 cycles with each cycle consisting of 5 mins at 94°C, 5 mins at 36°C and 5 mins at 72°C, and (ii) 30 cycles with each cycle consisting of 1 min at 94°C, 1 min at 36°C, and 2 mins at 72°C, followed by a final extension step at 72°C for 10 mins. The RAPD products (one-ninth of each reaction mixture) were then separated on a 1.5% agarose gels at 90 V/cm for 2 hrs using 1 X TBE as the running buffer. Molecular size standards (VC1Kb ladder) (Fermentas, USA) were also included on the gels. The fingerprints were compared visually followed by analysis using the Gel Compare II V 4.0 software (Applied Maths, Kortrijk, Belgium).

## 3. Results

### 3.1. Antibiotic Sensitivity Testing

#### 3.1.1. Disk Diffusion

The results indicated that, of the 81* B. pseudomallei* isolates tested, six were found to be MDR (resistant to MERO, IMP, and CAZ). However, the remaining 75 isolates were resistant to at least one of the antimicrobial agents (non-MDR). The MDR isolates had susceptibility results that were distinct from other isolates tested (Table 2). The MERO- and IMP-resistant isolates had smaller inhibition zone (15.7 mm) compared to the sensitive isolates (20 to 25 mm). These isolates were also persistently mucoid and the inhibition zone diameter was not distinct. Among the 81* B. pseudomallei* isolates, only 4.94% were CAZR and 2.47% showed intermediate susceptibility to CAZ, which is among the drug of choice for treatment of melioidosis. The CAZR isolates had no inhibition zone compared to the CAZ-sensitive isolates (24 to 29 mm).

#### 3.1.2. MIC Etest

The highest reading for the Etest results (0.125–128 mg/L) was demonstrated for the MICs of CAZ, which also correlated with the most resistant isolate identified by disk diffusion (Tables 2 and 3). The Etest results also demonstrated that 100% of the CAZR isolates were shown to be resistant to MERO. The isolate with the highest measurable CAZ Etest result was also found to confer highest resistance towards MERO.

#### 3.1.3. MIC Broth Microdilution

Conventional broth microdilution MIC results for CAZ, AMC, MERO, IMP, and TS were found to correlate with the Etest MIC results (Table 3). However, there was no significant correlation between MICs of MERO Etest and MICs determined by the disc diffusion and Etest.

#### 3.1.4. PCR Amplification

**Figure 1:**
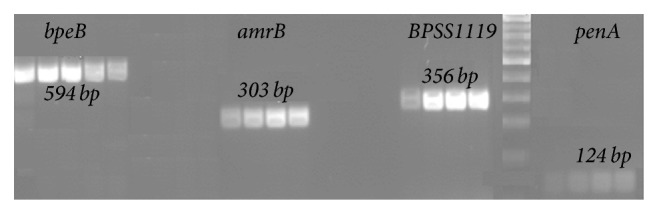
PCR results demonstrating the agarose gel electrophoretic analysis of PCR products generated from amplification of efflux pump genes (*bpeB*,* amrB*, and* BPSS1119*) and class A *β*-lactams gene (*penA*) from* B. pseudomallei*. The presence of a 594 bp, 303 bp, 356 bp, and 124 bp fragments in the agarose gel indicated a positive result for* bpeB*,* amrB*,* BPSS1119*, and* penA*, respectively. Ladder: 100 bp ladder (MBI Fermentas, USA).

PCR results demonstrated that the efflux pump genes (*bpeB*,* amrB*, and* BPSS1119*) and class A *β*-lactams gene (*penA*) were present in all the six MDR isolates found in this study (Table 2, Figure 1). We also observed that isolates which showed the presence of* penA* also demonstrated the presence of* bpeB*,* amrB*, and* BPSS1119*.

#### 3.1.5. Molecular Typing of Antibiotic-Resistant* B. pseudomallei* Strains Using RAPD

**Figure 2:**
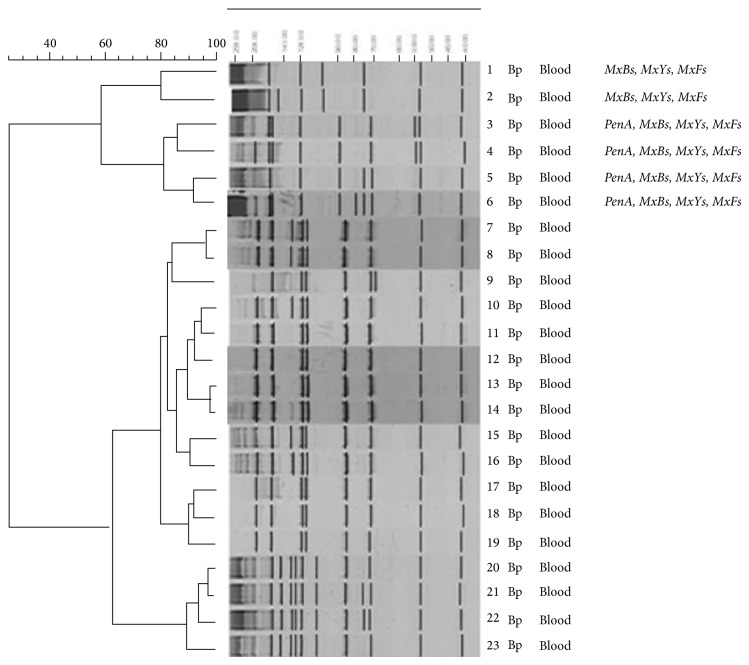
Random amplified polymorphic DNA (RAPD) polymorphisms of* B. pseudomallei* clinical isolates amplified by primer 272. The representative fingerprint patterns of two clusters RAPD were shown (i) RAPD cluster A (MDR) and (ii) RAPD cluster B (non-MDR).

A total of six different PCR fingerprinting patterns were generated from the six MDR isolates. However, eight different PCR fingerprinting patterns were generated for the remaining 75 non-MDR isolates. Based on the differences in the banding patterns, a distinct cluster (A) was identified among the 6 MDR isolates which contain the* bpeB*,* amrB*, and* BPSS1119* and* penA* genes, and among the 75 non-MDR isolates, only a single distinct cluster (B) was found (Table 2 and Figure 2).

## 4. Discussion 

In the current scenario, melioidosis is considered as a fatal disease with no effective vaccine available. Treatment also requires prolonged and high dosage antibiotic therapy to accomplish complete eradication. Nevertheless, prolonged therapy can lead to the development of resistance and to make matters worse, the causative agent,* B. pseudomallei,* is intrinsically resistant to a wide range of antibiotics. Thus, treatment options for melioidosis are limited to a small number of antimicrobial agents such as CAZ (primary treatment) and TS, DOXY, or AMC (secondary treatment) [21]. Despite many trials, CAZ remains as the drug of choice for treatment of severe melioidosis [22]. Carbapanems have also been shown to be highly active against* B. pseudomallei* [23]. As a result, resistance developed towards these antibiotics can pose a significant challenge in treatment of melioidosis.

In our study, comparison of Etest and broth microdilution against disk diffusion test for 81* B. pseudomallei* strains and a* B. thailandensis* strain showed broad range of MICs and zone inhibition, respectively. Our findings may also be indicative of the increased antibiotic resistance in* B. pseudomallei* clinical strains. In short, it appears that either disc diffusion or Etest could be applied for susceptibility testing in the case of CL, AMC, DOXY, MERO, IMP, CAZ, TGC, CLA, and TS in* B. pseudomallei* isolates (Table 2).

Additionally, in general, the Etest based antimicrobial susceptibility testing has the advantage of providing quantitative MICs results, which may be useful for clinicians to select appropriate treatments while the disc diffusion is a more reliable and cost effective technique for determining the prevalence of resistance among* B. pseudomallei* isolates in the more routine monitoring of melioidosis. A study by Piliouras et al., on the comparison of antibiotic susceptibility testing methods for TS with* B. pseudomallei*, also reported that the disc diffusion test was inappropriate for assessing the susceptibility of* B. pseudomallei* to TS. The study suggested that MIC based methods such as Etest may be a better choice for the determination of susceptibility for TS [24]. Difficulty in interpretation of disc diffusion results have also been implicated in previous studies which demonstrate varying susceptibility test results for TS with* B. pseudomallei* [25, 26]. This may be attributed to a few reasons including the imprecise endpoints for this combination of antimicrobial agent and organism as well as NCCLS guidelines being based on control standards of* Pseudomonas aeruginosa* and not* B. pseudomallei* [27, 28]. Thus, it is also evident that further creation of suitable internationally accepted MIC/zone inhibition breakpoints needs to be established through multilaboratory quality control. Several studies have been proposed for determination of the MICs, since these methods are cost effective and still able to produce reliable results [15, 29].

We also performed molecular level investigation to detect the presence of efflux pump and class A *β*-lactamase genes, which were found to be present in all the MDR* B. pseudomallei* isolates. Previous studies have associated the mutations in class A *β*-lactamase gene of* B. pseudomallei* to the resistance towards some cephalosporins and *β*-lactamase inhibitors [15]. It has been previously demonstrated that mutations in the* B. pseudomallei* class A *β*-lactamase coded by* penA* may confer resistance of the organism to CAZ. Both the low and high level of CAZR in the study of* B. pseudomallei* clinical isolates were confirmed by the detection of* penA* gene [15]. The presence of* penA* in* B. pseudomallei* used in our study matched the disc diffusion and MICs results.

Additionally, the necessity to characterise genes encoding for MDR efflux pump arises from the increased resistance of* B. pseudomallei* to a number of antibiotics. The presence of resistant nodulation division (RND) efflux pumps may contribute to the acquired resistance to fluoroquinolones and cross-resistant to unrelated antimicrobials [30]. This is not surprising as the efflux pumps can be associated with MDR isolates since they may be specific for one substrate or transport a wide range of antibiotics of multiple classes [31]. Primers* MxBs*,* MxYs*, and* MxFs* were used to amplify genes that encode proteins which are parts of the RND efflux pump [30]. The role of this efflux pump is associated with resistance towards a wide range of antibiotics including pefloxacin, ofloxacin, and CAZ in* B. pseudomallei* and accompanied by an increased resistance to aminoglycosides, *β*-lactams, macrolides, and CL [30, 31]. In this study, isolates that contained these genes (*bpeB*,* amrB*, and* BPSS1119*) were shown to be resistant to AMC, CLA, and CAZ as reported using the disc diffusion and MICs. The* bpeB*,* amrB*, and* BPSS1119* genes used in our study focus on chromosomally encoded MDR efflux pumps, which have been the best developed and the most widely used for demonstrated efflux pump genes using primers [31].

In conclusion, our study demonstrated the antimicrobial susceptibility profiles of* B. pseudomallei* against antibiotics that are commonly used for treatment including CAZ, AMC, DOXY, and TS as well as other antimicrobials such as CL, MERO, IMP, TGC, and CLA. The antimicrobial susceptibility profiles did not show any significant correlation with the origin of the isolates as similarly reported in a previous study [32]. We also highlighted that the CAZR is due to the presence of* penA*,* bpeB*,* amrB,* and* BPSS1119* in* B. pseudomallei*. However, except for the six blood isolates, none of the animal, soil, sputum, pus, lung, or spleen isolates were tested positive with the PCR detection assays for* penA*,* bpeB*,* amrB*, and* BPSS1119*. Thus, it may be possible that the resistant blood isolates were from relapse patients or patients who did not comply with antibiotic treatment. However, this remains to be elucidated. Horizontal dissemination of these genes may contribute to further emergence of CAZR in various other Gram-negative bacteria. Therefore, appropriate surveillance and control measures are essential to prevent further spread of the CAZR organisms. Further study using PCR-single-stranded conformational polymorphism (PCR-SSCP) to identify the point mutations which take place in the* B. pseudomallei* isolates will be used to compliment and facilitate better understanding of the impact of CAZR in clinical practice.

## Figures and Tables

**Table 1:** Primers used in this study.

Name	Target gene	Sequence	Reference
PenA-F	*pen A *	5′GTTCAGCAGATCTAACAGATCGCCGAGATGG3′	[15]
PenA-R		5′GCACCGCGATATCTCGCGCTCCGTGAACCTT3′	

MxBs-F	*bpeB *	5′GATCTCCGCGCAGAACGT3′	[16]
MxBs-R		5′AGGCCGATCGCGAGCACG3′	

MxYs-F	*amrB *	5′TTCATGCAGAACTTCCGC3′	[16]
MxYs-R		5′GAACGCCATCGGCACGAA3′	

MxFs-F	*BPSS1119 *	5′CGTCGTGATTTTCCTGTT3′	[16]
MxFs-R		5′ACGCGAACTCCTTGAACA3′	

272	Variable	5′AGCGGGCCAA3′	[17]

**Table 2:** Origin, resistogram (disk diffusion), and RAPD of 81 *B. pseudomallei* isolates and a *B. thailandensis* isolate.

StrainID	Origin of isolation^a^		Antimicrobial susceptibility testing						PCR detection			
		CL	CLA	DOXY	IMP	MERO	TGC	TS	CAZ	AMC	*PenA, bpeB, amrB* & *BPSS1119 *	RAPD
Bp1	Blood	I	S	S	S	S	R	S	S	S	Negative	B
Bp2	Blood	I	I	S	S	S	R	S	S	S	Negative	B
Bp3	Blood	I	I	R	R	S	R	S	S	I	Negative	B
Bp4	Blood	S	I	S	S	S	R	S	S	R	Negative	B
Bp5	Blood	R	R	R	R	R	R	R	R	R	*PenA, bpeB, amrB* & *BPSS1119 *	A
Bp6	Blood	S	S	S	S	S	R	S	S	I	Negative	B
Bp7	Blood	I	S	S	S	S	R	S	S	R	Negative	B
Bp8	Blood	S	S	S	S	S	I	S	S	S	Negative	B
Bp9	Blood	R	R	S	S	S	R	S	S	R	Negative	B
Bp10	Blood	S	S	S	S	S	R	S	S	R	Negative	B
Bp11	Urine	S	S	S	S	S	R	S	S	R	Negative	B
Bp12	Blood	I	S	S	S	S	S	S	S	I	Negative	B
Bp13	Blood	S	S	S	S	S	S	S	S	I	Negative	B
Bp14	Blood	S	S	S	S	S	S	S	S	S	Negative	B
Bp15	Blood	S	I	S	S	S	S	S	S	I	Negative	B
Bp16	Sputum	I	I	S	S	S	R	S	S	I	Negative	B
Bp17	Blood	I	S	S	S	S	R	S	S	I	Negative	B
Bp18	Pus	R	I	R	R	R	R	R	R	R	*PenA, bpeB, amrB* & *BPSS1119 *	A
Bp19	Blood	I	I	S	S	S	R	S	S	S	Negative	B
Bp20	Blood	S	I	S	S	S	R	S	S	I	Negative	B
Bp21	Blood	I	S	S	S	S	R	R	S	R	Negative	B
Bp22	Blood	S	S	S	S	S	R	R	S	S	Negative	B
Bp23	Blood	I	I	S	S	S	R	I	S	I	Negative	B
Bp24	Lung	S	S	S	S	S	R	I	S	I	Ngative	B
Bp25	Blood	I	R	R	R	R	R	R	I	R	*PenA, bpeB, amrB* & *BPSS1119 *	A
Bp26	Blood	S	S	S	S	S	S	S	S	I	Negative	B
Bp27	Blood	S	S	S	S	S	S	R	S	I	Negative	B
Bp28	Blood	S	I	S	S	S	S	I	S	I	Negative	B
Bp29	Blood	I	S	S	S	S	S	R	S	I	Negative	B
Bp30	Spleen	I	S	S	S	S	S	S	S	I	Negative	B
Bp31	Blood	S	S	S	S	S	R	R	S	I	Negative	B
Bp32	Blood	I	I	S	S	S	R	I	S	R	Negative	B
Bp33	Blood	I	I	S	S	S	S	R	S	I	Negative	B
Bp34	Blood	S	I	S	S	S	S	I	S	S	Negative	B
Bp35	Blood	R	R	R	R	R	S	R	I	S	*PenA, bpeB, amrB *&* BPSS1119 *	A
Bp36	Blood	S	S	S	S	S	S	S	S	S	Negative	B
Bp37	Blood	R	I	R	R	R	R	R	R	I	*PenA, bpeB, amrB*, & *BPSS1119 *	A
Bp38	Blood	I	I	S	S	S	R	I	S	S	Negative	B
Bp39	Blood	S	S	S	S	S	R	S	S	S	Negative	B
Bp40	Blood	S	S	S	S	S	R	S	S	S	Negative	B
Bp41	Sputum	I	S	S	S	S	R	S	S	R	Negative	B
Bp42	Blood	I	I	S	S	S	S	I	S	I	Negative	B
Bp43	Blood	I	I	S	S	S	S	S	S	I	Negative	B
Bp44	Blood	I	I	S	S	S	S	S	S	I	Negative	B
Bp45	Blood	I	I	S	S	S	S	S	S	S	Negative	B
Bp46	Blood	I	R	S	S	S	S	S	S	I	Negative	B
Bp47	Blood	S	S	S	S	S	R	I	S	R	Negative	B
Bp48	Blood	I	R	S	S	S	S	S	S	I	Negative	B
Bp49	Blood	S	I	S	S	S	R	S	S	R	Negative	B
Bp50	Blood	I	I	S	S	S	R	I	S	R	Negative	B
Bp51	Blood	S	S	S	S	S	R	I	S	R	Negative	B
Bp52	Blood	R	R	S	S	S	R	S	S	R	Negative	B
Bp53	Blood	I	R	S	S	S	R	I	S	I	Negative	B
Bp54	Blood	I	R	S	S	S	S	S	S	R	Negative	B
Bp55	Blood	R	S	S	S	S	S	I	S	I	Negative	B
Bp56	Blood	I	I	S	S	S	S	I	S	R	Negative	B
Bp57	Pus	I	R	S	S	S	S	I	S	R	Negative	B
Bp58	Sputum	S	S	S	S	S	S	R	S	R	Negative	B
Bp59	Sputum	S	I	S	S	S	R	I	S	I	Negative	B
Bp60	Sputum	S	S	S	R	R	R	R	S	R	Negative	B
Bp61	Animal	S	I	S	S	S	S	I	S	S	Negative	B
Bp62	Animal	I	S	S	S	S	I	I	S	R	Negative	B
Bp63	Animal	S	S	S	S	S	S	I	S	R	Negative	B
Bp64	Animal	S	S	S	S	S	S	S	S	S	Negative	B
Bp65	Animal	I	S	S	S	S	I	S	S	R	Negative	B
Bp66	Animal	I	S	S	S	S	I	S	S	S	Negative	B
Bp67	Animal	S	S	S	S	S	S	S	S	I	Negative	B
Bp68	Animal	I	S	S	S	S	R	S	S	R	Negative	B
Bp69	Animal	I	S	S	S	S	S	S	S	S	Negative	B
Bp70	Animal	I	S	S	S	S	I	S	S	S	Negative	B
Bt71	Soil	I	I	S	S	S	R	I	S	R	Negative	B
Bp72	Blood	I	I	S	S	S	R	S	S	R	Negative	B
Bp73	Blood	R	I	S	S	S	R	S	S	R	Negative	B
Bp74	Blood	I	R	S	S	S	R	I	S	R	Negative	B
Bp75	Blood	S	S	S	S	S	S	S	S	S	Negative	B
Bp77	Blood	R	R	S	S	S	R	S	S	S	Negative	B
Bp78	Blood	R	I	S	S	S	I	S	S	S	Negative	B
Bp79	Blood	R	R	I	S	S	R	I	R	R	*PenA, bpeB, amrB *&* BPSS1119 *	A
Bp80	Blood	R	R	I	S	S	R	I	S	I	Negative	B
Bp81	Blood	I	S	S	S	S	R	S	S	I	Negative	B
Bp82	Soil	I	S	S	S	S	R	S	S	R	Negative	B

Foot note: R: resistant; I: intermediate; S: sensitive; CL: chloramphenicol; AMC: amoxiclin/clavulanic acid; DOXY: doxycycline; MERO: meropenem; IMP: imipenem; CAZ: ceftazidime; TGC: tigecycline; CLA: clarithromycin; TS: trimethoprim/sulfamethoxazole.

Intermediate is counted as resistant.

^a^Blood, pus, sputum, urine, spleen, and lungs are of human origin.

Bp: *B. pseudomallei*; Bt: *B. thailandensis*.

**Table 3:** Broth microdilution and Etest MICs of 81 *B. pseudomallei* isolates.

Antimicrobial agent	MIC (mg/L)					
	Broth dilution			Etest		
	Range	50%	90%	Range	50%	90%
Chloramphenicol	4–16	8	16	0.25–24	3	8
Amoxiclin/clavulanic acid	4–8	8	8	0.125–2	0.25	0.25
Doxycycline	0.5–1	0.5	1	0.19–16	0.19	16
Meropenem	3-4	2	4^a^	1–4	1	1.5
Imipenem	0.5–1	0.5	1^a^	0.094–8	0.094	0.125
Ceftazidime	2–64	2	4^a^	0.125–128	0.250	0.5
Tigecycline	3-4	2	6	0.5–32	3	6
Clarithromycin	4–16	4	16	1.5–48	1	32
Trimethoprim/sulfamethoxazole	4–64	16	64	0.003–0.25	0.032	0.125

Foot note: ^a^multidrug resistant isolates.
